# Graphene Coating Obtained in a Cold-Wall CVD Process on the Co-Cr Alloy (L-605) for Medical Applications

**DOI:** 10.3390/ijms22062917

**Published:** 2021-03-13

**Authors:** Łukasz Wasyluk, Vitalii Boiko, Marta Markowska, Mariusz Hasiak, Maria Luisa Saladino, Dariusz Hreniak, Matteo Amati, Luca Gregoratti, Patrick Zeller, Dariusz Biały, Jacek Arkowski, Magdalena Wawrzyńska

**Affiliations:** 1 Division of Optical Spectroscopy, Institute of Low Temperature and Structure Research, Polish Academy of Sciences, Okólna 2, PL-50-422 Wrocław, Poland; lukaszwasyluk76@gmail.com (Ł.W.); v.boiko@intibs.pl (V.B.); m.markowska@intibs.pl (M.M.); d.hreniak@intibs.pl (D.H.); 2 Carbonmed Spółka z ograniczoną odpowiedzialnością, ul. Okólna 2, PL-50-422 Wrocław, Poland; dariusz.bialy@umed.wroc.pl (D.B.); jacek.arkowski@umed.wroc.pl (J.A.); 3 Institute of Physics of the National Academy of Science of Ukraine, Prospect Nauky 46, UA-03028 Kyiv, Ukraine; 4 Department of Mechanics, Materials Science and Biomedical Engineering, Wrocław University of Science and Technology, Smoluchowskiego 25, PL-50-370 Wrocław, Poland; mariusz.hasiak@pwr.edu.pl; 5 Department of Biological, Chemical and Pharmaceutical Sciences and Technologies (STEBICEF) and INSTM UdR—Palermo, University of Palermo, Viale delle Scienze, Bld. 17, IT-90128 Palermo, Italy; marialuisa.saladino@unipa.it; 6 Elettra–Sincrotrone Trieste S.C.p.A., SS14—km 163.5 in Area Science Park, IT-34149 Trieste, Italy; matteo.amati@elettra.eu (M.A.); luca.gregoratti@elettra.eu (L.G.); patrick.zeller@posteo.de (P.Z.); 7 Helmholtz-Zentrum Berlin für Materialien and Energie GmbH, BESSY II, Albert-Einstein-Straße 15, DE-12489 Berlin, Germany; 8 Fritz-Haber-Institut der Max Planck Gesellschaft, Dept. Inorganic Chemistry, Faradayweg 4-6, DE-14195 Berlin, Germany; 9 Department of Preclinical Research, Faculty of Health Sciences, Wroclaw Medical University, Ludwika Pasteura 1, PL-50-367 Wrocław, Poland

**Keywords:** graphene coating, biocompatibility, cobalt-chromium alloy, cold-wall chemical vapor deposition method

## Abstract

Graphene coating on the cobalt-chromium alloy was optimized and successfully carried out by a cold-wall chemical vapor deposition (CW-CVD) method. A uniform layer of graphene for a large area of the Co-Cr alloy (discs of 10 mm diameter) was confirmed by Raman mapping coated area and analyzing specific G and 2D bands; in particular, the intensity ratio and the number of layers were calculated. The effect of the CW-CVD process on the microstructure and the morphology of the Co-Cr surface was investigated by scanning X-ray photoelectron microscope (SPEM), atomic force microscopy (AFM), scanning electron microscopy (SEM), and energy dispersive X-ray spectroscopy (EDS). Nanoindentation and scratch tests were performed to determine mechanical properties of Co-Cr disks. The results of microbiological tests indicate that the studied Co-Cr alloys covered with a graphene layer did not show a pro-coagulant effect. The obtained results confirm the possibility of using the developed coating method in medical applications, in particular in the field of cardiovascular diseases.

## 1. Introduction

Graphene is one of the most prospective materials in terms of its unique properties for many applications in the field of electronics [1,2] electricity [3], sensors [4], biosensors [5], catalysis, etc. [1,2,3,6]. At the same time, there are several scientific reports on the use of graphene-based materials in biology and medicine [7,8,9]. One of the ideas is the utilization of its chemically inert properties for coating medical devices and the use of the external surface as a biologically neutral protective anti-corrosion film [10,11,12,13]. Covering implants and medical instruments with graphene-like materials does not lead to the problem of releasing graphene from the human body because the material is attached to the implant. However, for this particular case, the inertness of graphene leads to problems with coating attached to the substrate material; the coating technology must ensure that it is stable and tear-resistant in a biological environment. For effective metal surface coating, it is therefore necessary to prepare the surface of the substrate to be coated and to optimize the chemical vapor deposition (CVD) process, allowing better attachment, e.g., by using the defective interface side of the graphene layer for bonding. Additionally, the high temperatures required for the processes used for deposition of metallic coatings on 3D structures (e.g., scaffolds) are problematic in many cases. This problem becomes critical for alloys, mainly due to the danger of overheating the material (microstructural changes, recrystallization) and possible phase transitions affecting its mechanical properties. In this work, we assumed that these changes can also lead to weakening of the alloy–graphene interactions. To date, a large area of high-quality and uniform graphene grown by the CVD method was obtained for Cu or Ni foils [14,15]. However, the CVD method is also used for the graphene growth on Co [16], Fe [17], Pt [18], and other transition metal substrates [19] or their oxides, including co- or tri-metallic alloys. It has been demonstrated and intensively studied in thousands of papers [19,20]. Besides, a modified cold-walled CVD (CW-CVD) method has been successfully used to obtain high-quality graphene layer for Cu [21,22], cobalt [16], and cobalt-nickel alloy [23]. However, the same coating on the complex, modern medical alloys has not been reported yet, and it still is a technological challenge. In this work, for the first time, a successful graphene-coating of Co-Cr medical alloy, one of the materials commonly used for cardiovascular applications, is presented [24]. In our previously reported research, we showed that graphene coating obtained by poly(methyl methacrylate) (PMMA) method promotes vascular endothelial cells growth on the covered surface and thus shows promising biocompatibility features for cardiovascular applications [25]. For investigated materials, we paid attention to two main aspects affecting the harmfulness of the proposed solution: (1) homogeneity of coating and its mechanical stability on the alloy surface (possibility of de-attaching of graphene flakes) and (2) chemical interaction of tissues with graphene, especially at the interface of graphene and alloy substrate where some chemical bonding between graphene and alloy may be expected—probably via oxygen, forming graphene oxide (GO) regions. In both aspects, tentative answers were provided by the hemocompatibility tests we carried out.

## 2. Results and Discussion

### 2.1. Optimization of the Deposition Process

The obtained alloy discs were subject to the graphene coating procedure, optimized by changing the parameters of the CW-CVD process. The initial set of deposition process parameters was based on the results described by Macháč et al. [16] who successfully used the CW-CVD process to prepare graphene on pure cobalt substrates. The first experiments were conducted for both polished and unpolished alloy substrates. However, since Raman spectroscopy used as the first probe testing for graphene confirmed its existence only on unpolished samples, optimization of the procedure and related characterization were performed only for these samples. Optimal results were obtained by subjecting unpolished Co-Cr substrates to the CW-CVD process consisting of four stages: heating (SP1), annealing in argon with hydrogen to remove the oxygen residues at the sample surface (SP2), subsequent heating (SP3), and the stage of graphene formation and growth (SP4) (Table 1).

In order to obtain the optimal parameters for the best quality graphene layer, a series of samples was prepared. At the stage of graphene formation and growth (SP4), the time (1800, 2700 and 3600 s) and the deposition temperature (800, 900 and 1000 °C) changed. At the final stage, the cooling process was supplied in an argon flow atmosphere up to 100 °C.

### 2.2. Structure Characterization by SEM and EDS Method

**Figure 1:**
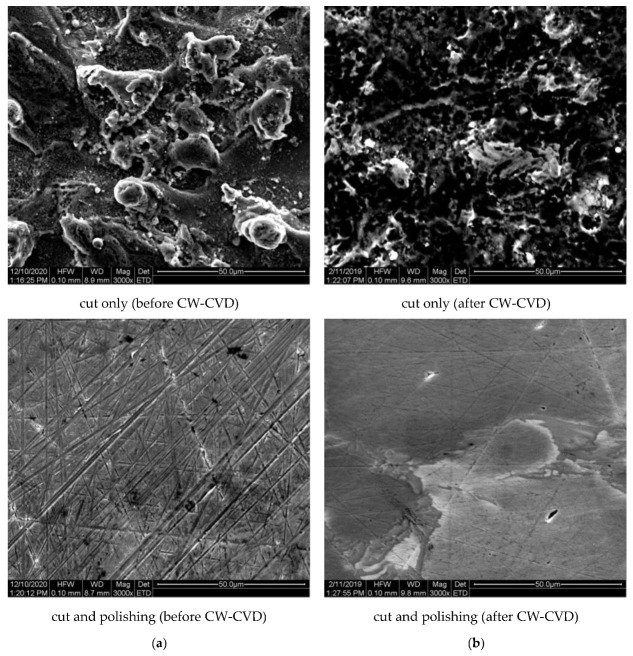
SEM micrographs cut (top) and polished (bottom) sample before (**a**) and after (**b**) CW-CVD.

SEM and energy dispersive X-ray spectroscopy (EDS) analyses were carried out for all the alloy samples obtained. The investigations performed allowed us to visualize the influence of surface preparation of the alloy samples on their morphology. Examples of SEM micrographs made for cut and polished surfaces and cut only samples obtained after CW-CVD processing are shown in Figure 1a,b, respectively. As it can be seen, the surfaces of samples differed significantly. The polished sample, apart from the obvious polishing marks, was characterized by the disappearance of all structures on the surface, while the sample without polishing was characterized by an extended surface, indicating a strong influence of spark cutting on the separation of different alloy fractions.

**Figure 2:**
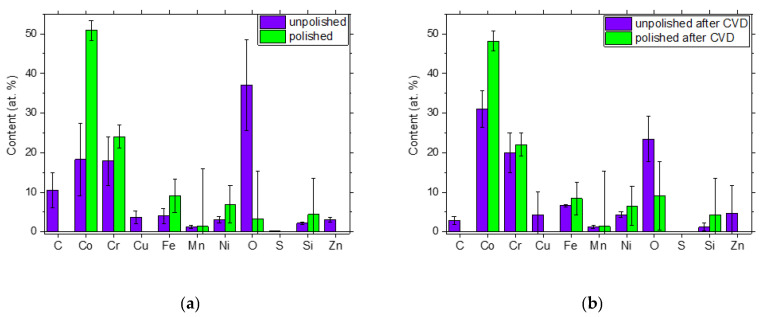
The averaged results of the energy dispersive X-ray spectroscopy (EDS) analysis performed for Co-Cr alloys before (**a**) and after graphene deposition (**b**).

On comparison of the SEM micrographs before and after the CW-CVD process, a slight homogenization of the surface microstructure was also evident for both unpolished and polished samples, manifesting itself in a slight decrease in the roughness of the unpolished sample and the disappearance of scratches on the polished sample. Additional information is provided by the results of EDS microanalysis performed on the chosen area of these samples (Figure 2). It can be seen, among other things, that polishing had a fundamental influence on the oxygen content of the analyzed surface. This was manifested by the almost ten times higher oxygen content for the unpolished sample compared to the polished one. It can also be seen that subjecting the same sample to an effective graphene coating in the CW-CVD process resulted in only a slight change in the oxygen content, still much higher than for the polished surface. Observed difference between the C content between the sample before and after polishing (Figure 2a) was most probably due to presence of M6C carbides on the surface of the as-cut sample. It is known that carbide precipitates (grain-boundary carbide precipitates [26]) are often present after heat treatment of this type of alloy. The applied polishing of the disc surface may have led to their removal from the surface, which was reflected in the values determined from the EDS. Additionally, since carbon was present in a wide range in the surrounding environment, and the samples were attached to a conductive tape containing carbon during the measurement, it seems that the measurement of the carbon percentage may have been disturbed because of this. Additionally, the identification of carbon content is always subject to a large measurement error due to the low energy on the EDS spectrum compared to the other elements present in the alloy under study. However, the percentage content with respect to the measurement error of the other elements in the alloy was not questionable.

**Figure 3:**
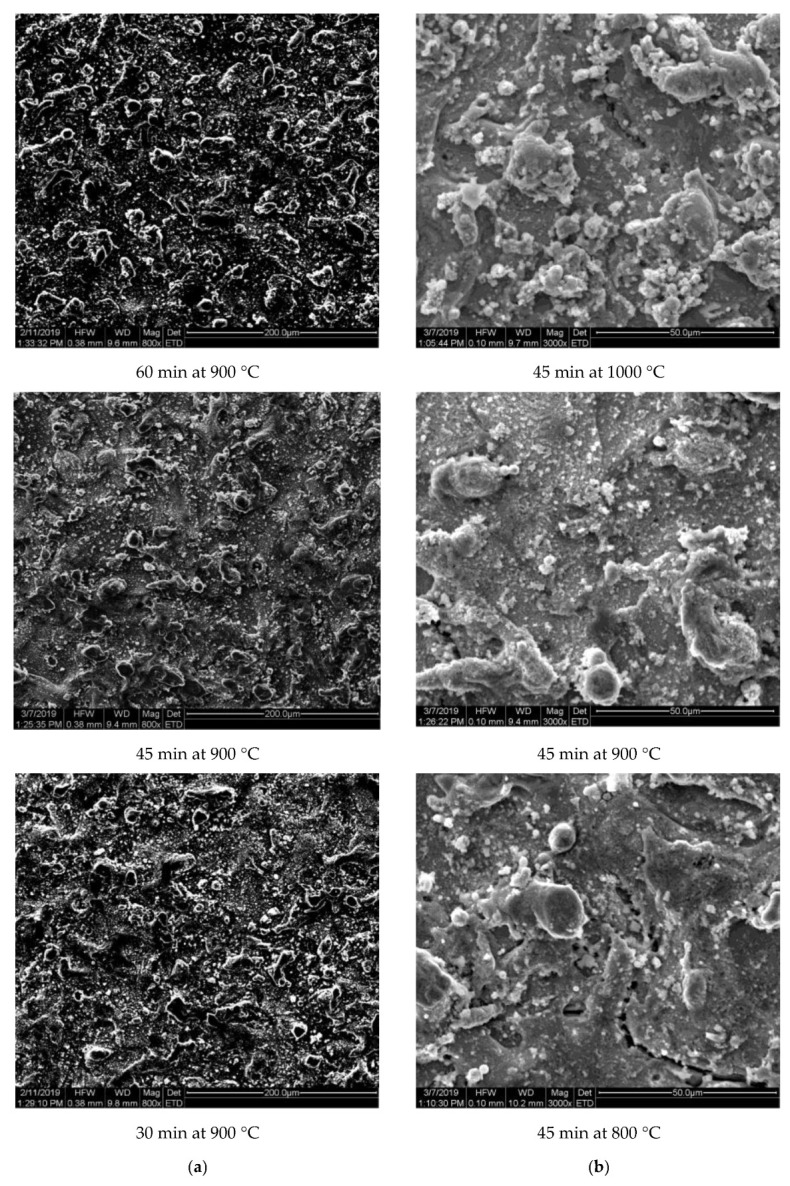
SEM micrographs of Co-Cr alloys after CW-CVD as function time deposition (**a**) and temperature deposition (**b**).

Additional analyses with the SEM micrographs were carried out to establish the influence of modification of the CW-CVD application parameters for non-polished samples with the graphene layer (Figure 3).

As we can see from the micrographs shown in the left panel (Figure 3a), the surface microstructure did not change noticeably with a change in the duration of the process at 900 °C. On the other side, we see some modification of the structure with the temperature increase from 800 °C to 1000 °C (Figure 3b). Most of the few micrometer sized irregular cracks in the surface of the alloy evident in the case of sample after the processing at 800 °C for 45 min disappeared after being treated at 900 °C.

### 2.3. Investigations of Topography by Atomic Force Microscopy (AFM)

**Figure 4:**
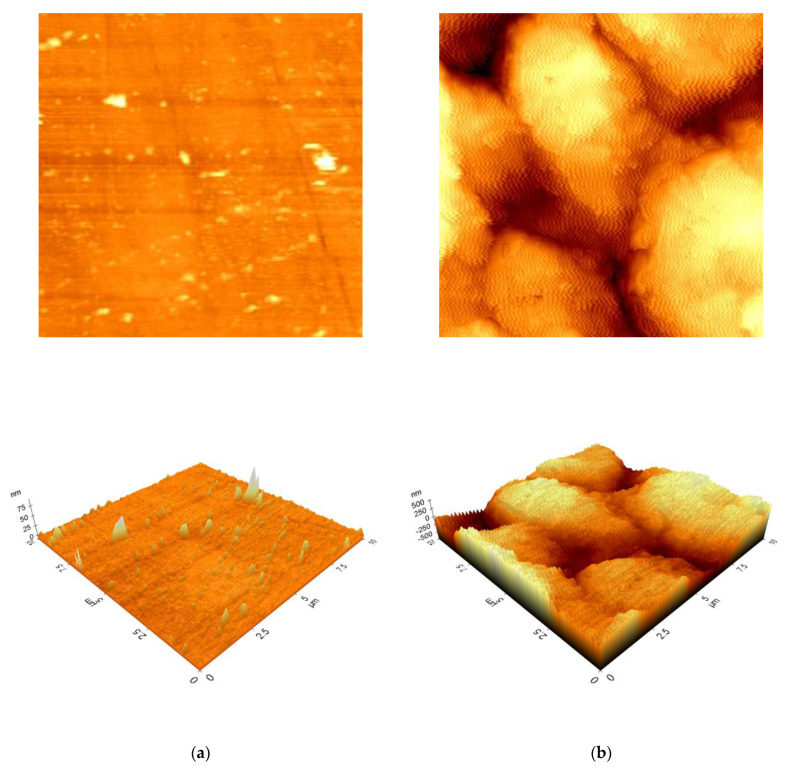
2D/3D atomic force microscopy (AFM) topography images for polished (**a**) and cut after CW-CVD process (**b**) samples of Co-Cr alloy recorded in contact and noncontact mode, respectively.

One of the most advanced, versatile, and powerful methods is the measurement of the surface topography on the atomic scale by atomic force microscopy. These investigations deliver 2D/3D topography images as well as various types of surface characterization. The 2D and 3D topography images recorded for samples of Co-Cr alloy polished and cut after CW-CVD process are presented in Figure 4.

It is well visible that the topography of the cut sample after CW-CVD process in comparison to the polished sample was not homogeneous, and some features of about 2–3 µm width were present. The difference between the minimum and the maximum values in the recorded surface profile equaled 1273.09 nm. The root mean-squared roughness (Rq), the average roughness (Ra), and the ten point average roughness (Rz) for the image presented in Figure 4a were 215.18 nm, 175.89 nm, and 1241.08 nm, respectively. The complex surface of cut samples allowed us only to record the topography of the surface in noncontact mode. The polished samples of Co-Cr alloy presented quite different surface structure, and only a few objects less than 1 µm wide were registered in the topography scanning process. This measurement was performed in contact mode, which allows for a much more accurate projection of the analyzed surface. The difference between the minimum and the maximum values in the recorded surface profile equaled 101.24 nm, and it was 12.6 times lower than for cut samples. The roughness parameters Rq, Ra, and Rz for the surface presented in Figure 4b were 4.32 nm, 2.27 nm, and 88.54 nm, respectively.

### 2.4. Raman Spectroscopy

**Figure 5:**
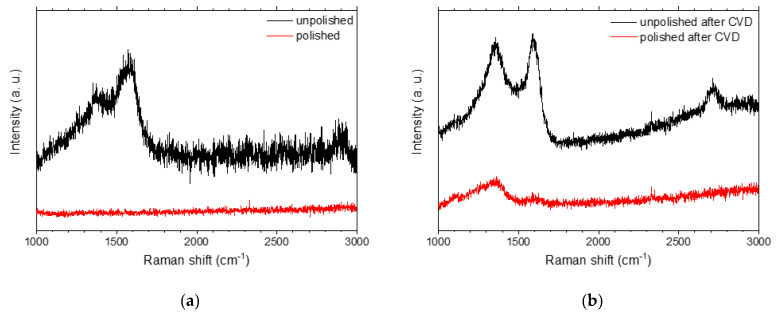
Raman spectra of the unpolished (cut only) and the polished Co-Cr alloy samples (before (**a**) and after CW-CVD (**b**)).

For each deposited sample, the presence of the G band and the quality of the graphene layer were tested by Raman spectroscopy. The presence of such a coating consisting of several overlapping layers of graphene was evidenced by the presence of characteristic peaks for graphene: D, G, and 2D peaks. They were located at around 1350 cm^−1^, 1580 cm^−1^, and 2700 cm^−1^, respectively [27,28]. The G peak was a result of stretching vibrations of the sp^2^ pairs in both rings and chains, while the D peak was due to the breathing modes of the sp^2^ pairs in rings. The 2D peak was the second order of the D peak [29]. Preliminary measurements of the samples after the CW-CVD process for graphene deposition showed a complete lack of specific bands on each polished substrate (Figure 5). Meanwhile, for the unpolished sample before CW-CVD process, two broad covered bands around 1500 cm^−1^ that can be related to low sp^3^ amorphous carbon were present [29].

**Figure 6:**
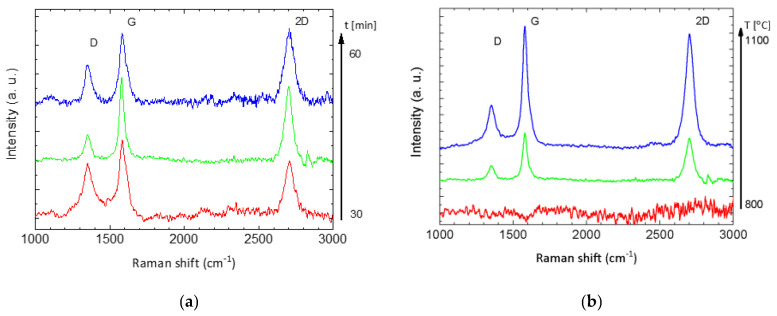
Raman spectra of the Co-Cr alloys as a function of the time deposition (**a**) and the temperature deposition (**b**).

Therefore, in order to understand the factors influencing the quality of the graphene layer and to optimize the deposition process, spectra of samples obtained with different CW-CVD parameters were recorded only for non-polished samples. Representative Raman spectra of the samples obtained in both (temperature and time) series are shown in Figure 6.

**Figure 7:**
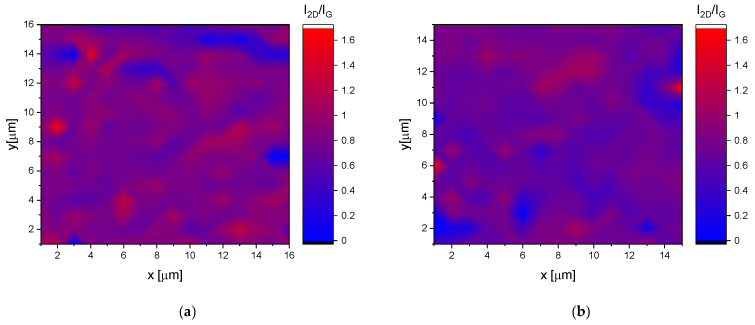
Map made on the basis of Raman spectra collected for a sample deposited at 900 °C (**a**) and 1000 °C (**b**).

As shown above, the optimal process parameters (t = 2700 s, T = 900 °C) yielded the best results in terms of design and quality of layers. The half-widths of 2D, G, and D bands were 55.5, 45, and 53 cm^−1^, and the ratios of the intensity of the 2D and the D bands to the G band were 0.94 and 0.35. Both with shorter and longer deposition times of the graphene layer growth stage, more defective layers were obtained, as evidenced by a more intense D peak in relation to the G peak intensity (Figure 6a). At a temperature lower than 900 °C, no graphene layer was formed at all (red line in Figure 6b), while at 1000 °C, a graphene layer was formed, but the sample surface coverage was incomplete, which is clearly visible in Figure 7 below, where the areas are marked with blue color, in which no trace of graphene is present (the ratio of 2D peak intensity to G goes to zero).

The shape and the intensity of the 2D peak were significantly different in graphene compared to bulk graphite. This is because, in bulk graphite, the 2D peak consisted of two components, while graphene had a single, sharp 2D peak with Lorentzian shape [29]. Its intensity, compared to the G band, could vary from three to one-third from graphene to graphite, respectively. When disorder increased, this band broadened, overlapping with other bands, and nearly disappeared. The relative intensity ratio between the 2D and the G bands was also found to be dependent on the number of layers. I_2D_/I_G_ was close to three for monolayer graphene and fell to 0.3 for highly oriented pyrolytic graphite [30].

According to the shape of the 2D band and the ratio of its intensity to the intensity of the G band, the number (up to five) of the graphene layers and their quality could be clearly identified [27]. The intensity ratio (I_2D_/I_G_) maps based on the Raman data were constructed (Figure 7). As it can be seen, for alloys with graphene deposited at 900 °C (Figure 7a), the intensity ratio maps had a more homogenous color compared to alloys with graphene deposited at 1000 °C (Figure 7b). The discontinuities in the graphene layer (blue areas), together with a sharper color gradient from point to point, may indicate an increase of disorder in the graphene layers.

### 2.5. Mechanical Properties

**Figure 8:**
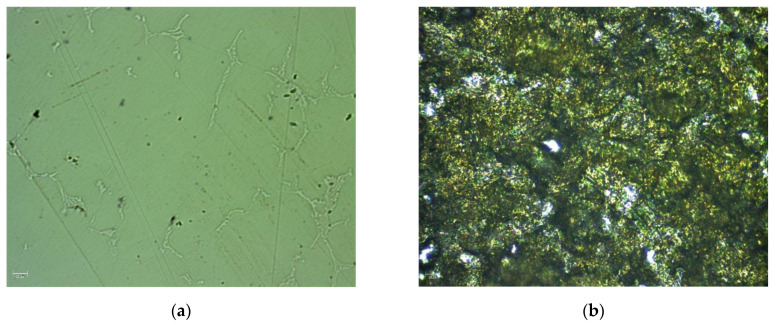
Microscope images recorded in polarized light for polished (**a**) and unpolished (**b**) Co-Cr samples.

Mechanical properties of Co-Cr discs were investigated for polished and cut (after CW-CVD process) Co-Cr samples. Figure 8 shows the surface of these samples observed in polarized light. In the case of the polished sample, the grain boundaries represented by fine needles were clearly visible. For the cut sample, only a very complex structure was observed. These results are in good agreement with data obtained from AFM investigations, and they determined the mechanical parameters.

Instrumented nanoindentation is a measurement method that allows one to investigate the mechanical properties such as hardness, elastic modulus, and deformation energies of the samples where conventional Vickers hardness measurements cannot be performed due to the small sample dimension of the samples or low load. Therefore, the determination of parameters of mechanical properties for Co-Cr discs was performed by measurements of indentation depth as a function of applied load with respect to ISO 14577 standard.

**Figure 9:**
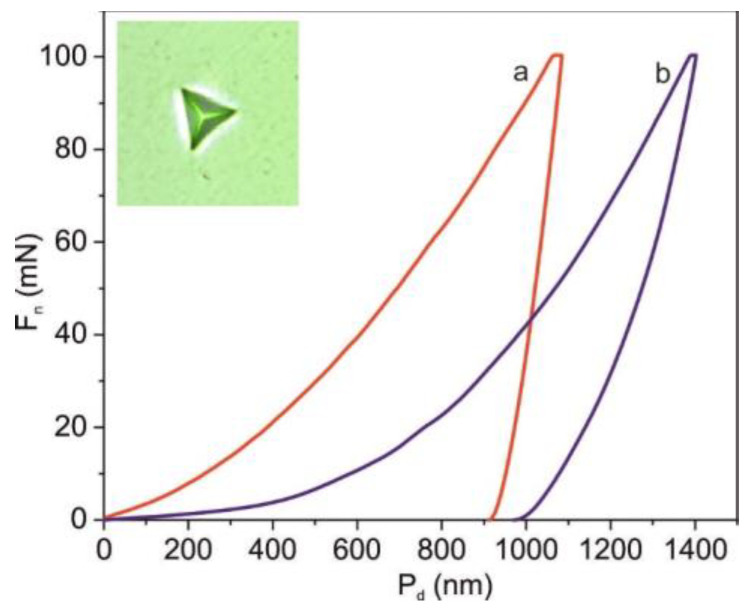
Example of load (F_n_) versus indentation penetration depth (P_d_) curves for polished (**a**) and cut after CW-CVD process (**b**) Co-Cr discs. The inset shows an imprint obtained for polished sample.

Figure 9 shows the dependence of penetration depth versus a maximum load of 100 mN for polished and cut Co-Cr samples. Moreover, an imprint of the Berkovich diamond indenter for the polished sample is also shown. The results obtained from numerical analysis of F_n_-P_d_ curves with respect to the Oliver–Pharr procedure are presented in Table 2. It can be seen that instrumental hardness, instrumental elastic modulus, as well as plastic and total deformation energies of the polished sample were higher than for the cut samples. This change was related to the formation of new structures on the disk surface during the CVD process.

**Figure 10:**
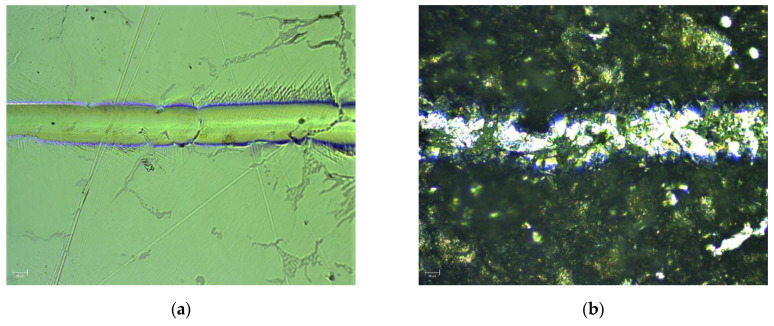
Microscopic image of the residual groove after a scratch test on polished (**a**) and unpolished (**b**) Co-Cr disc.

To determine the quality of Co-Cr disc surface, the scratch tests were performed for polished and cut samples. The pictures of the surface obtained for the investigated materials are presented in Figure 10.

**Figure 11:**
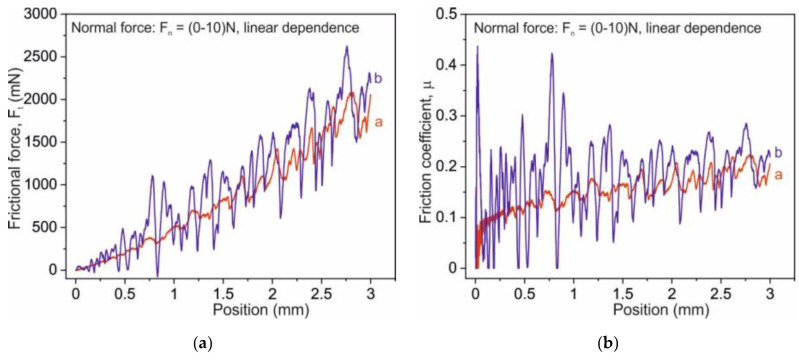
Frictional force displacement curves for scratch tests (left side) and friction coefficient displacement curves for polished (curve **a**, red color) and cut (curve **b**, blue color) Co-Cr discs.

Numerical analyses of the frictional force and the friction coefficient for the polished and the cut Co-Cr discs are presented in Figure 11. All measurements were performed for a length of 3 mm and a maximum load of 10 N.

It is seen that frictional force and friction coefficient increased with the load applied during measurement for both materials. The large fluctuations of frictional force and friction coefficient (for low load) recorded for cut samples were mostly caused by surface unevenness. Moreover, with increasing normal force, an increase in F_t_ and µ was observed for both samples.

### 2.6. Scanning X-Ray Photoelectron Microscope (SPEM)

**Figure 12:**
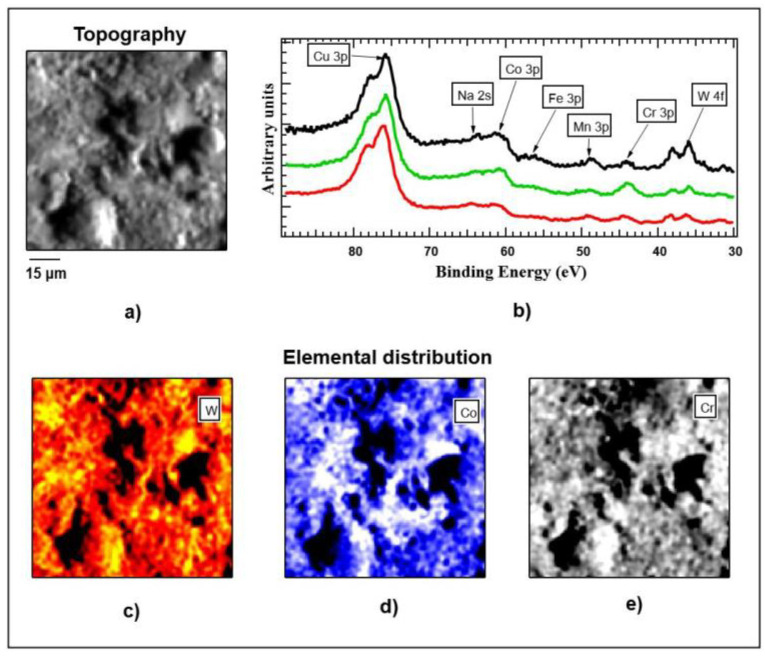
Scanning X-ray photoelectron microscope (SPEM) map of the unpolished sample covered by graphene. The contrast present in the image is dominated by surface topography (**a**) brighter areas are associated with features which are oriented towards the hemispherical electron energy analyzer (HEA), while dark regions represent shadowed areas or holes in the surface. X-ray photoelectron spectroscopy (XPS) spectra (**b**) acquired at different representative positions. (**c**–**e**)—elemental distribution of W, Co, and Cr, respectively. The black areas visible in the images must be ignored because of the intensity of the photoemission signal detected from these areas, which is too poor to be correctly processed. Changes in the color scale reflect the variations in the elemental concentration.

The image shown in Figure 12 is a SPEM map of the unpolished sample covered by graphene. The contrast present in the image is dominated by surface topography; brighter areas are associated with features oriented towards the hemispherical electron energy analyzer (HEA) while dark regions represent shadowed areas or holes in the surface.

**Figure 13:**
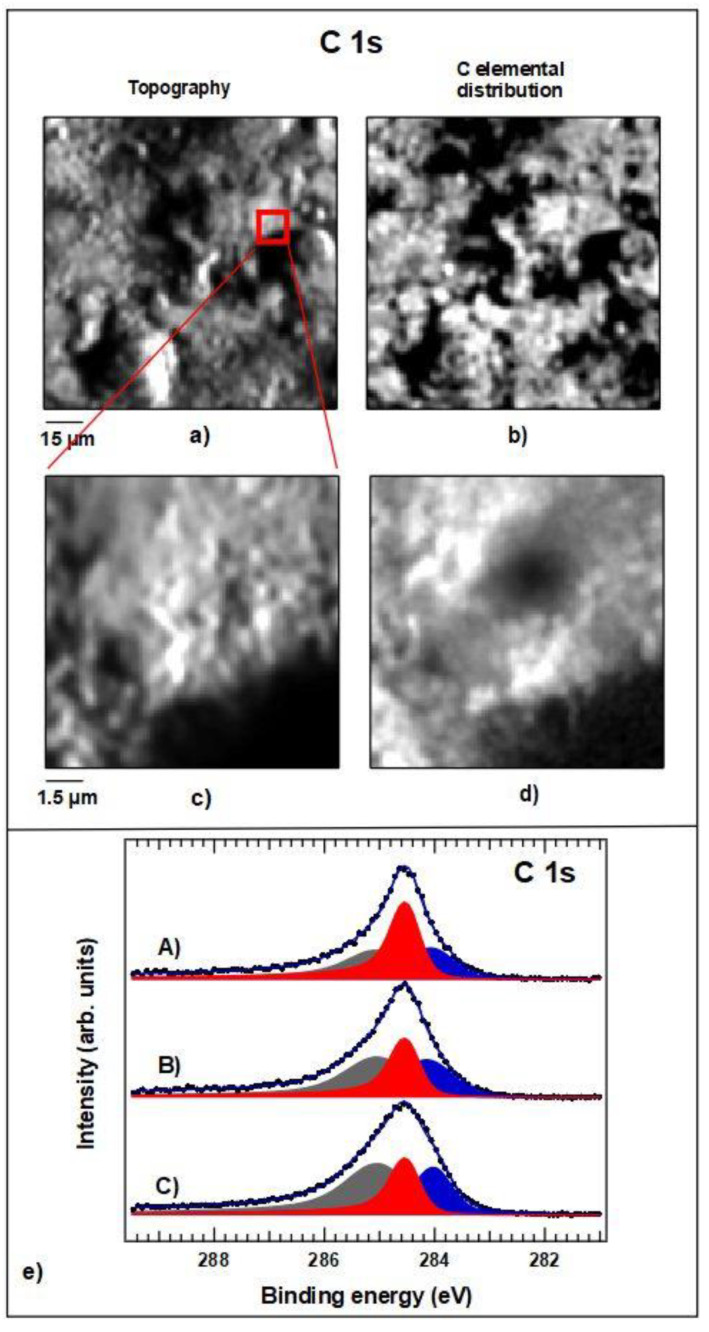
(**a**,**b**) raw data SPEM image acquired at the C 1s level and the corresponding C elemental distribution. (**c**)—same maps of the magnified region indicated by the red square in image and (**d**) same maps of a magnified region after processed. (**e**) representative C 1s spectra acquired at different locations. The three color filled lines are the result of the deconvolution procedure, which separates the contribution of the different chemical C moieties. See text for the details.

As a result, the raw data images acquired at different elements appeared similar. A collection of XPS spectra acquired at various regions of the map in Figure 12 are reported in Figure 13; the core levels of distinct elements were present, namely Cu 3p, Na 2s, Co 3p, Fe 3p, Mn 3p, Cr 3p, and W 4f, as indicated in the graph.

The extended surveys (not shown here) additionally reported the presence of C and traces of Si. The three spectra shown in the panel were normalized with respect to the Cu 3p intensity for a better comparison; the relative intensities of W, Mn, Cr and Co changed in each spectrum, suggesting a different chemical composition in the probed areas. To investigate deeper the elemental heterogeneity of the surface, we processed the raw data maps acquired at the different core levels and removed the topographic contribution of the contrast, highlighting the elemental one according to well-known algorithms, as described in [31]. The results of this analysis for W, Co, and Cr are shown in the three maps of Figure 12c–e, respectively. The black areas visible in the images must be ignored because of the intensity of the photoemission signal detected from these areas, which is too poor to be correctly processed. Changes in the color scale reflect the variations in the elemental concentration, supporting what is evidenced by the three spectra of Figure 12b. The distribution of the concentration of W showed variations as high as 80% in the different areas, while those associated with Co and Cr did not exceed 30%. The list of detected elements reflected well the EDS data reported in Figure 2. It is important to note that SPEM data reflect the chemical composition of the outermost atomic layers of the surface, since the escape depth of photoelectrons was limited to 1–1.5 nm. Such thickness, including the graphene coating, will be analyzed later. The XPS spectra revealed a high surface concentration of Cu. This was not a surprise, since EDS analysis showed the presence of Cu only in the unpolished samples, suggesting a segregation of Cu in the outermost areas, which were removed with polishing. The BEs of the core levels detected agree with high oxidation states of the same as expected for such alloy.

The distribution of carbon over the surface is shown in Figure 13a,c are the raw data maps recorded at two different levels of magnification. Additionally, in this case, the majority of the contrast in the pictures was generated by the topography of the surface. By applying the same processing algorithms as done for the maps in Figure 6, we isolated the local elemental concentration and show it in maps Figure 13b,d, respectively. In agreement with the Raman micrographs of Figure 5 and Figure 6, the distribution of C appears not uniform at both magnification scales. For this reason, it is not straightforward to evaluate the thickness of the Gr–C layer, but we can state that all spectra that were acquired at different regions allowed the detection of the substrate elements, indicating an overall thickness of the C coating of less than 1–1.5 nm. To investigate more in detail the chemical state of the C coating, high resolution XPS spectra of the C 1 s were acquired at different areas. Three representative spectra are shown in Figure 13e; the three color filled lines are the result of the deconvolution procedure, which separated the contribution of the different chemical C moieties. For this procedure, we used Doniac-Sunjich functions, which describe the photoemission process and are commonly used for XPS peaks deconvolution. The red component centered at 285.5 eV BE shows the typical line shape generated by the graphene two-dimensional layers of carbon atoms with sp^2^ hybridization that were connected in a hexagonal lattice; as expected, this component was narrower than the other ones. The blue filled line centered at 284.0 eV BE was typically associated with defects in the C hexagonal lattice of the graphene, while the broad grey filled one at 285.0 eV BE took into account all other C-C, C-O, and organic carbon species, which generally adsorb on a surface. The weights of the components in the three spectra were different, proving that the chemical state of carbon was heterogeneous over our surface; in some areas, the graphene was predominant, while in others, adventitious carbon was more abundant.

### 2.7. Biocompatibility Studies

#### 2.7.1. Genotoxicity Studies

The tested extracts from Co-Cr discs coated with graphene did not show any mutagenic effect on TA98 and TA100 strains, both used separately and in combination with the S9 fraction.

The tests also showed that the application of the polar (−FBS) and the non-polar (+FBS) solutions did not cause significant differences in the appearance of reversion. The number of reverse wells in all tested strains after incubation with extracts from Co-Cr discs coated with graphene was less than 10. There was also no effect of direct and inversely proportional formation of a greater number of reversions with increasing concentration of the examined extracts. The test was performed in triplicate.

#### 2.7.2. Cytotoxicity Studies

Cytotoxicity studies showed that the extracts of the Co-Cr discs tested showed low toxicity, which was estimated at the level of one according to the cytotoxicity scale. Extracts from Co-Cr discs coated with graphene showed little toxicity only at the highest concentration of 100% used, and at the remaining concentrations tested of 50%, 25%, and 12.5%, no toxicity was observed against mouse fibroblast cells. Moreover, the use of the serum extract (FBS +) or without (FBS −) showed no significant differences in cytotoxicity. Detailed results of cytotoxicity tests are presented in Table 3.

**Figure 14:**
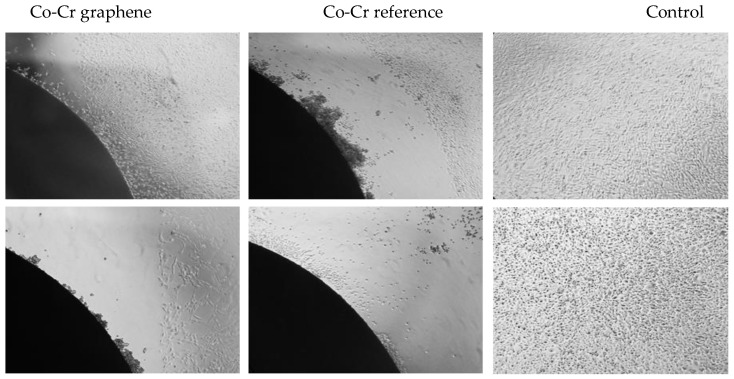
Evaluation of direct contact of BALB/3T3 mouse fibroblast cells with the examined discs. Co-Cr disks are visible as a dark semi-circle in the lower left corner, negative control high-density polyethylene (HDPE) of cells without Co-Cr disks. Magnification 50×.

The tests carried out on the discs in direct contact showed a moderate reactivity towards mouse fibroblast cells, estimated according to the reactivity scale at level three. Placement of Co-Cr discs on the BALB/3T3 cell monolayer (Figure 14) resulted in the appearance of shrunken cells around the disc and a free zone up to 1 cm. This effect was observed both in the case of the disc covered with a layer of graphene and the reference disc. In turn, sowing cells directly onto Co-Cr discs also resulted in the appearance of shrunken cells around the disc and a free zone up to 1 cm.

**Figure 15:**
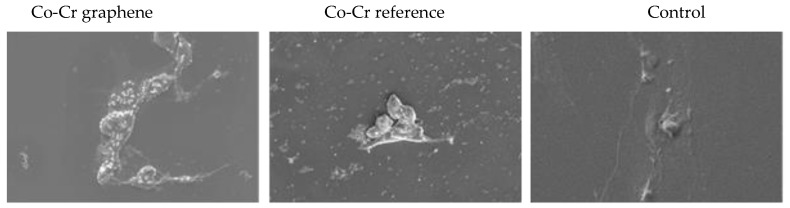
Evaluation of direct contact of BALB/3T3 mouse fibroblast cells with the examined discs. Deposits and cells are marked in orange. Magnification 2000×.

The surface of the tested Co-Cr discs after incubation with cells was also analyzed. It turned out that there were no cells on the surface of Co-Cr discs located on the cell layer of mouse fibroblasts. However, BALB/3T3 cells were seen on the surface of Co-Cr discs plated with cells. Cells were present on the surface of both the Co-Cr disc with a graphene layer and on the surface of the Co-Cr disc of the reference. It was noticed that the degree of cell surface coverage was higher in the Co-Cr disc without the graphene layer (Figure 15).

The tested extracts from Co-Cr discs coated with graphene and without a graphene layer showed no toxic effect on the cells of BALB/3T3 mouse fibroblasts. The highest applied concentrations caused a slight cytotoxic effect on a five-point scale, estimated at level one, while for in vitro cytotoxicity, cytotoxicity above grade two classifies an extract as toxic.

In direct contact tests, both Co-Cr discs with and without a graphene layer resulted in the appearance of shrunken cells and a free zone around the disc up to 1 cm, which was assessed on a five-point scale at level three as moderate reactivity. One of the reasons for this reactivity may be the fact that the discs were not permanently attached to the bottom of the plate and could move slightly, causing mechanical damage to the cells. In contrast, the results obtained using the scanning electron microscope showed that, in the wells with Co-Cr discs, there were lumpy structures and deposits that adhered to the cell surface, which could cause a toxic effect. The analysis of the elemental composition of EDS confirmed that these were exfoliating fragments of the Co-Cr discs tested. Interestingly, the analysis of the surface of the examined discs after incubation with the cells showed that the cells of the murine BALB/3T3 fibroblasts attached and grew on the surface of both types of Co-Cr discs coated and uncoated with graphene. This observation suggests that the surface of the discs was not toxic to the cells, while the fragments exfoliating from it caused a cytotoxic effect.

#### 2.7.3. Hemocompatibility Studies

**Figure 16:**
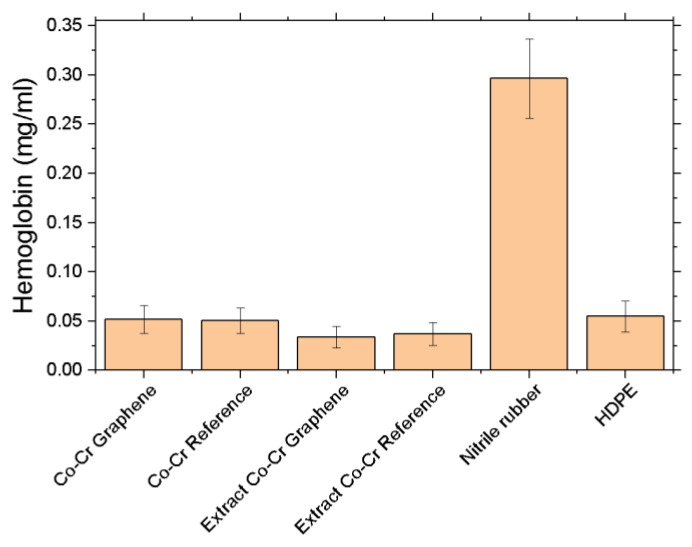
Hemoglobin level after incubation with test materials and extracts from Co-Cr discs.

Hemolysis assay: Incubation of tested Co-Cr discs with a concentrate of red blood cells did not cause hemolysis. In the material after incubation with Co-Cr discs with and without graphene, about 0.05 mg/mL of hemoglobin was observed. On the other hand, when red blood cells were incubated with extracts, a slightly lower level of hemoglobin, approximately 0.03 mg/mL, was recorded. The positive control, which was nitrile rubber, caused a significant increase in the level of hemoglobin to the level of approximately 0.3 mg/mL compared to the other tested groups. High-density polyethylene (HDPE) was not observed to significantly increase hemoglobin levels. This level was similar to the level observed after incubation with Co-Cr disks and disk extracts (Figure 16).

Clotting time and coagulation factors measurements: Evaluation of the activated partial thromboplastin time (APTT) showed that none of the materials tested increased the APTT. In all studied groups, an increase in fibrinogen levels was observed, but differences in these results were not statistically significant between the groups. No differences were observed for other parameters such as prothrombin time, thrombin time, and recalcination time. Tests of the activity of blood clotting inhibitors such as ATIII showed that, in all the trials, the level of this factor was increased compared to the prescribed norm. However, it was not observed that the differences in ATIII level between the samples were statistically significant. There were no differences between the samples in the activity of plasminogen and protein C. The study of coagulation factors VII, X, and XII showed that neither the tested Co-Cr discs nor the extracts obtained from these discs caused statistically significant changes in the level of the factors.

**Figure 17:**
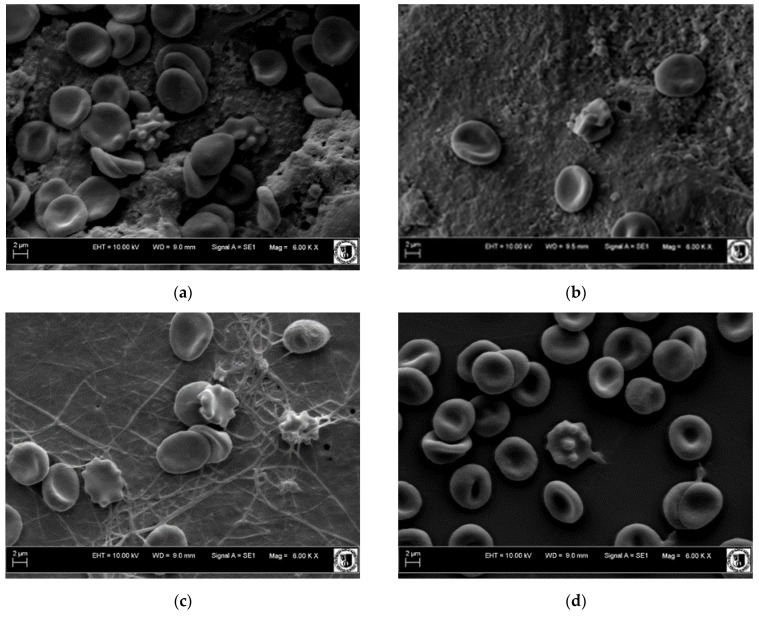
Surface of tested materials after contact with solid blood. Co-Cr disc with graphene (**a**), Co-Cr disc without graphene (**b**), nitrile rubber (**c**), high-density polyethylene (HDPE) (**d**). Magnification 6000×.

Blood platelets activation analysis: The analysis of the surface of the tested materials showed that, after incubation with whole blood, all the materials had morphosis elements of blood in the form of red blood cells, white blood cells, and platelets. Red blood cells on all materials showed normal morphology. On the surface of the Co-Cr discs tested, no clot appeared. Numerous activated platelets were observed, but no clot formation was observed. According to the scale for clot formation, both Co-Cr discs with and without graphene layer obtained a result of zero. The situation was definitely different with the use of nitrile rubber fragments as a positive control, where strongly activated plates and clot assessed at level three were visible. For the formation of a clot on high-density polyethylene, a result of zero was obtained. Photos of the surface of the tested materials with blood morphotic elements are shown in Figure 17.

The conducted tests show that the tested Co-Cr discs with and without a graphene layer showed good hemocompatibility. No changes in the morphology of blood components were observed, and normal morphology of red blood cells was observed on the surface of the tested materials. Incubation of whole blood with the tested materials showed that they did not cause significant changes in the composition of morphotic elements. No differences were observed between the tested Co-Cr discs with and without graphene and the control materials in the form of nitrile rubber and high-density polyethylene.

## 3. Materials and Methods

### 3.1. Materials

The samples of substrates were prepared in the form of disks with a diameter of 7 mm and a thickness of ~1 mm. The discs were cut using an electrical discharge machining (EDM) with electric sparks underwater from an alloy sheet of a cobalt-based L-605 alloy of standardized composition: Co 55.76–65.193%, Cr 27–31%, Fe 2–4.5%, Ni 3%, Mo 0.8–1.5%, Si 0.5–1, 5%, C 0.8–1.4%, Mn 0.6–1.2%, Cu 0.08%, S 0.015–0.03%, P 0.012–0.03%. Before graphene deposition, the obtained samples were divided into two parts. One of them was additionally polished, and both of them were subjected to the following washing process (step by step):

- sonication in acetone (99.5% pure P.A.-Basic) for 10 min in an ultrasonic bath;
- sonication in ethyl alcohol (96% pure P.A.-Basic) for 10 min in an ultrasonic bath;
- sonication in isopropanol (99.7% pure P.A.-Basic) for 10 min in an ultrasonic bath.

The ultrasonic procedure permits efficient removal of all organic contaminations and also any residual of grease or oil coming from the stents production process.

### 3.2. Methods

#### 3.2.1. Cold-Wall Chemical Vapor Deposition (CW-CVD)

Deposition of graphene on the cobalt-chrome alloys was carried out by the cold-wall CVD method on the commercial nanoCVD-8G system from Moorfield Nanotechnology Ltd. (Manchester, UK). The alloy substrates were placed directly on the heater (4.0 cm × 2.5 cm) embedded thermocouple. Programmable logic controller electronics equipped with a touchscreen interface controlled the hardware, continuously reporting the reactor chamber state: heater and chamber temperatures, pressure, and gas flow. Maximum operating temperature was 1100 °C, and maximum process gas flow was: argon −200 sccm (standard cubic centimeters per minute); hydrogen −20 sccm; methane −20 sccm. Pressure parameters: capacitance manometer 20 Torr (full scale), and valid entries were in the range of 0.0–0.5 Torr.5.

#### 3.2.2. Raman Spectroscopy

Raman spectra were recorded using a Renishaw inVia Raman microscope (100×, magnification) equipped with Argon laser (λ_ex_ − 514 nm, power maximum at sample surface ~9 mW) and a CCD camera under environmental conditions (Renishaw plc, Wotton-under-Edge, Gloucestershire, UK). Detection range was 100–3200 cm^−1^. Maps of relative intensity values of specific graphene bands were plotted based on a set of Raman spectra recorded in an automatic regime with a step of 5 microns.

#### 3.2.3. Atomic Force Microscopy (AFM)

The topography of the cobalt-chrome alloy (L-605) (measured as a deflection of the cantilever in the vertical direction) was investigated in contact and noncontact modes with scanning frequency of 0.5 Hz by using an atomic force microscope (XE-100, Park Systems, Suwon, Korea). All measurements were performed by scanning an area of 10 µm × 10 µm. The obtained data were analyzed using XEI Software (Version 4.3.4) provided by the microscope’s manufacturer. The roughness parameters such as Rq (root-mean-squared roughness), Ra (roughness average), and Rz (ten-point average roughness) were calculated for recorded images.

#### 3.2.4. Scanning Electron Microscopy (SEM)

The surface of coated cobalt-chrome alloy (L-605) samples as well as the surface of reference uncoated L-605 samples were observed using a scanning electron microscope (Quanta 200 W, FEI). The magnification used was from 200× to 3000×, Wehnelt’s electrode voltage 25 kV, Hi-Vacuum mode. Chemical composition of surfaces was analyzed with EDS detector (EDAX Element with solid silicon nitride (Si3N4) window and non-nitrogen Silicon Drift Detector) and dedicated software (TEAM by EDAX). Samples were mounted on single plates, with double-sided carbon tape.

#### 3.2.5. Scanning X-ray Photoelectron Microscope (SPEM)

The scanning photoelectron microscope (SPEM) allows spatially resolved XPS measurements in the submicron scale [32,33]. SPEM measurements were performed by using the Escamicroscopy beamline at Elettra synchrotron facility (Trieste, Italy). Samples were annealed in vacuum at about 200 °C for 5 h to remove residual surface contamination.

Spatial resolution in the submicron scale was achieved by de-magnifying the X-ray beam with a zone plate (ZP), a Fresnel type lens, plus an order sorting aperture (OSA) to stop the higher order produced by the ZP. Spot sizes down to 130 nm were obtained. The X-ray illumination was perpendicular to the sample surface. The emitted photoelectrons were collected by a hemispherical electron energy analyzer (HEA) mounted at a 30° angle with respect to the sample plane to enhance the surface sensitivity of the measurement; a delay line detector grouped the detected electrons in 48 channels. The overall energy resolution was better than 300 meV. The photon energy (hv) used for the measurements was 644.5 eV.

#### 3.2.6. Mechanical Properties

The investigations of mechanical properties were performed as nanoindentation measurements of hardness, elastic modulus, and deformation energies by NHT2 Nanoindentation Tester (CSM Instruments, Needham, MA, USA) equipped with a Berkovich diamond indenter with total included angle of 142.3°. During the measurement, the following parameters were applied: linear loading, maximum load −100 mN, loading/unloading rate −200 mN/min, Poisson’s ratio −0.3. The scratch tester (CSM Instruments, Needham, MA, USA) was used to determine the quality of the polished and the cut Co-Cr discs surface. The following parameters were used for measurements: linear scratch, begin load −30 mN, end load −10 N, loading rate −4985. The Rockwell indenter was applied (diamond with the radius of 0.2 mm).

#### 3.2.7. Biocompatibility Study

Sample preparation: The samples of investigated material were prepared in accordance with the guidelines described in the PN-EN ISO 10993-12: 2012 Biological evaluation of medical devices Part 12: Sample preparation and reference materials standard. Co-Cr discs were tested in two adequate ways:

— through direct contact with the tested Co-Cr disc;
— using extracts from the tested Co-Cr discs.

Genotoxicity studies: For this purpose, the Ames test was used (in accordance with OECD guidelines). The bacterial gene mutation test was used (according to PN-EN ISO 10993-3: 2009 Biological evaluation of medical devices Part 3: Testing for Genotoxicity). The study was performed using the Ames MPF TM 98/100 test (Xenometrix, Allschwil, Switzerland) on two strains of *Salmonella typhimurium* TA98 and TA100, both used separately and in combination with the S9 fraction obtained from the livers of Sprague-Dawley rats. The 25-fold more concentrated extracts were used, which were then diluted to obtain concentrations of 100%, 50%, 25%, 12.5%, 6.25%, and 3.125%. The positive controls for TA98 and TA100 strains without S9 fraction were 2-nitrofluorene (2-NF) and 4-nitroquinoline N-oxide (4-NQO). In turn, for strains TA98 and TA100 with fraction S9, 2-aminoanthracene (2-AA) was the control.

Cytotoxicity studies: Cytotoxicity testing was performed in accordance with the PN-EN ISO 10993-5 (2009) Biological evaluation of medical devices Part 5: Tests for in vitro cytotoxicity. BALB/3T3 clone A31 (ATCC^®^ CCL-163 ™) mouse fibroblast cells grown in Dulbecco’s Modified Eagle’s Medium (DMEM) with 10% fetal bovine serum and 1% antibiotic at a final concentration of 1% (10,000 U/mL of penicillin) were used in the study. The extracts from the Co-Cr discs were tested in four concentrations: 100%, 50%, 25%, and 12.5%, by diluting them in the culture medium. The negative control was high-density polyethylene (HDPE, Greiner, Rastatt, Germany), as a positive control, sodium lauryl sulphate solution (SLS, Sigma Aldrich, St. Louis, MO, USA) was used at concentrations of 0.2, 0.1, 0, 05, or 0.025 mg/mL, dissolved in the culture medium. The culture plates were incubated for 24 h. After this time, the morphology of the cells was assessed using a Leica DMi 1 light microscope (Leica, Wetzlar, Germany). Cytotoxicity was assessed using the MTT assay. For this purpose, the test solutions were removed from the wells of the culture plates, and the MTT reagent was added. Cells were then incubated for 2 h, after which time, the MTT solution was removed, and isopropanol was added to each well. After 30 min, a reading was made at a wavelength of 570 nm using an Epoch 2 spectrophotometric reader (BioTek, Highland Park, MI, USA). Cell viability was assessed using the formula: V = (Ab: As) × 100%, where V is cell viability, Ab is mean absorbance value in the test sample, and As is mean absorbance value in the blank sample.

Direct contact test was performed by two methods. The first method was to place sterile Co-Cr disks in separate wells at the bottom of the wells in a 24-well plate (Greiner, Rastatt, Germany) and then seed the cells onto Co-Cr disks. The second method involved seeding the cells, and after sticking to the bottom of the well and reaching 80% confluence, Co-Cr discs were placed on them. In both methods used, the number of cells plated per well was comparable.

After 24 h of incubation, the cell morphology was assessed using a Leica DMi 1 light microscope (Leica, Wetzlar, Germany), and then the cells and the Co-Cr discs were fixed with 2.5% glutaraldehyde (Avantor Performance Materials Poland SA, Gliwice, Poland) for 1 h. After fixation, cells were dehydrated in an increasing series of ethyl alcohol (from 50–100%). It was dried and coated with a 20 nm gold layer using a Leica EM ACE200 vacuum sputter sputtering machine (Leica, Wetzlar, Germany) and imaged on an Evo LS15 scanning electron microscope (Zeiss, Oberkochen, Germany).

All reagents were from Sigma Aldrich, St. Louis, MO, USA. Cells were grown under standard conditions at 37 °C in the presence of 5% CO2 in a Midi40 incubator (Thermo Fisher Scientific, Waltham, MA, USA).

Hemocompatibility studies: All adequate hemocompatibility testing was performed in accordance with PN-EN ISO 10993-5 (2009): Biological evaluation of medical devices Part 4: Selection of tests for interactions with blood. The performed tests included: hematological tests (assessment of number and morphology of blood cell elements), assessment of hemolytic activity, tests of platelet dysfunction, tests of clotting system activation (determination of activated partial thromboplastin time, prothrombin time, thrombin time, fibrinogen concentration, recalcination time), the activity of coagulation factors (VII, X, XII), activation of the fibrinolysis system (determination of fibrinolysis time, plasminogen concentration), testing of coagulation inhibitors (ATIII activity, protein C). Whole blood collected for EDTA and sodium citrate (BioIVT, West Sussex, Great Britain) and concentrated red blood cells (HaemoScan, Groningen, the Netherlands) were used in the research. Co-Cr discs with a total area of 3 cm^2^ were incubated with 1 mL of whole blood and incubated for 60 min at room temperature. Nitrile rubber materials (HaemoScan, Groningen, The Netherlands) with a total area of 0.5 cm^2^ were a positive control. The negative control was high-density polyethylene (HDPE, Greiner, Rastatt, Germany). Extracts from the tested Co-Cr discs in the volume of 300 µL were also used for the research.

After the incubation time elapsed, the blood counts were measured, and activated partial thromboplastin time, prothrombin time, thrombin time, fibrinogen concentration, recalcination time, level of clotting inhibitors (ATIII activity, protein C), and plasminogen activity were determined. The analyses were performed using a hematological and biochemical analyzer (Diagnostyka, Wrocław, Poland).

## 4. Conclusions

Samples with a layer of graphene throughout the large area of the Co-Cr alloy substrate (L-605) by a cold-wall CVD process were obtained and confirmed by the Raman spectroscopy measurements. It was shown by surface microanalysis studies that the surface of unpolished substrate samples with the demonstrated presence of graphene was not homogeneous and had still a relatively high average roughness compared to polished substrates. At the same time, measurements of the samples after the CVD process showed a complete absence of specific graphene bands on the polished Co-Cr substrates. This may indicate a critical influence of the preparation method, the resulting uniformity of the microstructure, and the chemical composition on the effectiveness of the graphene deposition process. The investigation of mechanical properties showed that instrumental hardness and elastic modulus of polished and cut only Co-Cr discs were equal to 406.31 Vickers, 161.49 GPa and 262.82 Vickers, 72.264 GPa, respectively. The results of the hemocompatibility test, understood as the ability to inactivate blood coagulation processes, indicate that Co-Cr alloy samples covered with a graphene layer did not show the pro-coagulant effect. The tested material also showed good biocompatibility. This fact, together with a proof of deposition of good quality layers by use of the relatively cheaper technique (CW-CVD), gives hope for the implementation of the developed technology in the field of coating medical devices.

## Figures and Tables

**Table 1:** Stages and corresponding process parameters in the cold-wall chemical vapor deposition (CW-CVD) chamber.

	t, s	T, °C	p, Torr	Ar, % (sccm)	H_2_, % (sccm)	CH_4_, % (sccm)
SP 1	0	90	10	5 (100)	1 (20)	0 (0)
SP 2	300	90	10	5 (100)	1 (20)	0 (0)
SP 3	0	900	10	5 (100)	1 (20)	0 (0)
SP 4	2700	900	10	0 (0)	2 (1.2)	35 (20)

t—time in second; T—heater substrate temperature in °C; sccm—standard cubic centimeters per minute.

**Table 2:** Mechanical properties as instrumental hardness (HV_IT_), instrumental elastic modulus (E_IT_), elastic deformation energy (W_elast_), plastic deformation energy (W_plast_), total deformation energy (W_total_), and elastic deformation energy to total energy ratio W_elast_/W_tot_ (n_IT_) for Co-Cr discs.

	Co-Cr (Polished Disc)	Co-Cr (Cut after CVD Process Disc)
HV_IT_ (Vickers)	406.31	262.82
HV_IT_ (MPa)	4387.30	2837.90
EIT (GPa)	161.49	72.26
W_elast_ (pJ)	6777.72	15,125.11
W_plast_ (pJ)	36,322.14	24,542.69
W_total_ (pJ)	43,099.86	39,667.80
n_IT_ (%)	15.73	38.13

**Table 3:** Evaluation of the cytotoxicity of extracts from tested Co-Cr disks, extract preparation solutions, and experiment control.

Sample	Concentration [%]	Cell Viability [%]	The Degree of Cytotoxicity
Co-Cr Graphene FBS+	100	77.09	1
	50	93.13	0
	25	98.36	0
	12.5	108.7	0
Co-Cr Graphene FBS−	100	81.12	1
	50	96.75	0
	25	94.32	0
	12.5	98.04	0
Co-Cr Reference FBS+	100	78.92	1
	50	83.59	1
	25	93.69	0
	12.5	91.46	0
Co-Cr Reference FBS−	100	84.12	1
	50	87.63	1
	25	89.92	0
	12.5	96.54	0
Positive control	0.2	15.63	4
	0.15	18.32	4
	0.1	63.17	3
	0.05	82.15	1
Negative control	-	98.02	0

## Data Availability

Data sharing is not applicable to this article.
